# Process Evaluation of the Los Angeles Unified School District Nutrition Network

**Published:** 2008-03-15

**Authors:** Antronette K Yancey, Richard E Kratz, Ninez A Ponce

**Affiliations:** Department of Health Services, University of California, Los Angeles, School of Public Health; Department of Family Medicine, University of California, Los Angeles, San Gabriel, California; School of Public Health, Department of Health Services, University of California, Los Angeles, Los Angeles, California

## Abstract

**Introduction:**

This study evaluated the Los Angeles Unified School District Nutrition Network, a large multicomponent nutrition and physical activity program in an ethnically diverse school district, launched in 2000.

**Methods:**

We calculated descriptive statistics and performed hierarchical logistic regression on school-level demographic and implementation data.

**Results:**

Thirty-six percent of eligible schools participated in 2001, and 79% of participating schools reapplied the following year. Elementary schools and schools that applied for grant money were more likely to reapply. Produce sampling was the most frequently cited program highlight, and making purchases with program grant money was the most frequently cited challenge.

**Conclusion:**

Our findings suggest that schools serving students of low socioeconomic status and diverse ethnicities can be recruited into a large program to promote healthy dietary choices and physical activity, especially elementary schools. Effectiveness and institutionalization of the program might be positively affected by fostering local ownership, allowing school personnel who apply for the grant to tailor the program to their individual schools.

## Introduction

The number of overweight children in the United States has continued to increase during the past several decades (1). The prevalence of overweight in young people aged 2 to 19 years increased 182% between 1971 and 2000, and the extent of overweight, that is the amount by which children are overweight, increased 247%, indicating overweight children are also becoming heavier (2). National data show that the prevalence of overweight in the non-Latino black and Latino adolescent populations was particularly high in 2000, reaching 23.6% and 23.4%, respectively (1). Between 1971 and 2000, the extent of overweight in young people aged 12 to 19 years had increased 292% in non-Latino blacks and 271% in Latinos in comparison with the 165% increase in non-Latino whites (2). This disproportional representation of obesity in ethnic minorities has produced an effort to focus on the trends, disparities, and approaches to overweight and associated diseases in children and adolescents in these groups (3).

Overweight prevalence among Los Angeles County public school children is estimated to be 26%, with another 19% of children at risk for becoming overweight (4). The Los Angeles Unified School District (LAUSD) enrolls 53% of the county's 1.7 million school children and has a high percentage of Latino (72%), and African American (12%) students, two of the ethnic groups most at risk for overweight (5). In response, the Los Angeles Unified School District Nutrition Network (LAUSDNN) was launched in 2000 with a grant from the California Nutrition Network for Healthy, Active Families. The funds originate from the U.S. Department of Agriculture and are intended to assist low-income, food-stamp–eligible households in adopting healthy eating habits and active lifestyles (6).

LAUSDNN is a school-based demonstration project that promotes vegetable and fruit consumption and increased physical activity. In the 2001–2002 school year, more than 200 schools participated in LAUSDNN, with the direct involvement of 6853 teachers, 174 food service workers, 195 administrators, and 117 school nurses. LAUSDNN made its resources available to schools with kindergarten through grade 12 (K–12), who were eligible to participate if they enrolled a majority of low-income students, defined as having 50% or more students who were eligible for free or reduced-price meals through the National School Lunch Program.

The major components of LAUSDNN were Harvest of the Month, the Harvest of the Month newsletter, action grants, the Chefs in the Classroom program, nutrition advisory councils, school gardening support, and individual school-generated activities. Schools applied for funding and participated in the program on a voluntary basis. Letters announcing availability of the program and funding were sent to eligible schools. LAUSD hosted a fall kick-off event at which the programs (e.g., Harvest of the Month, gardening) were announced. School personnel submitted "action grant" proposals, which were likely to involve classroom-based educational and behavioral curricula, but also may have included environmental changes, parental involvement, or community involvement. Allowable action grant expenditures on physical activity included teacher guides and media encouraging physical activity. Purchase of exercise equipment was not allowed (7). School personnel administering action grants were required to log hours spent on grant-related activities, but at the time of our study, there was no formal tracking of adherence to the grant proposals. LAUSDNN designed and directly administered all other components of the program.

Nutrition networks such as LAUSDNN generally function analogously to the service-oriented tobacco-use prevention efforts of California and other states, notably Oregon. In these states, the departments that control cigarette sales tax revenue have established mechanisms for providing competitive grants for tobacco use prevention to local agencies, including school districts (8-11). Locally developed, prospective grant programs are required to include evidence-based core principles to increase the likelihood of effectiveness. Depending on the state, these core principles may parallel the Centers for Disease Control and Prevention's guidelines (10-12) or may be more extensively tailored by the state (9-13). Both California and Oregon mandate performance evaluation for accountability and refinement of projects, making allowances for local flexibility (10-12).

Although school-based curricula alone may not prevent tobacco-use initiation (9-13), in these 2 states, comprehensive school-based efforts funded by competitive grants have shown progress toward this end. Students in schools funded by Oregon's Tobacco Prevention Education Program were 20% less likely to smoke than students at nonfunded schools after a 2-year exposure to the program (9). Evaluation of the California Tobacco Use Prevention Education competitive grant funding program found a significantly faster decrease in tobacco use in students in grant schools over the study period (14).

Despite the potential benefits of school-based nutrition demonstration projects, formal process evaluations of obesity prevention efforts in schools have largely been a part of multisite, randomized, controlled research initiatives (15-19). Generally these programs have centered on a formal classroom curriculum, but process evaluation lessons relevant to the present analysis are evident. For instance, the 5-A-Day Power Play Plus program received local producer support that provided fruits and vegetables for taste testing and home snack packs. Teachers rated these among the most effective parts of the curricula (15), and in one of the grade levels evaluated, outcome data showed fruit, juice, and vegetable consumption to be higher in the schools with more complete implementation of taste testing (20). In another 5-a-day program, 5-a-Day High 5, taste-testing stimulated high rates of student participation and enjoyment but was rated by teachers as among the most difficult activities to deliver. Additionally, process evaluation of this program found that the intervention was delivered less frequently and less consistently in schools with lower income families and larger African American enrollment (16). Gimme 5, another 5-a-day program, makes interpretation of process analysis results more difficult and casts some doubts on the validity of teacher self-reporting as an implementation process evaluation measure because of inconsistencies with observational data (17). The institutionalization process study arm of the Child and Adolescent Trial for Cardiovascular Health is relevant to this study because the investigators used a "manual approach" of data abstraction from interview questionnaires. Reviewers identified common themes from this extracted data. In analyzing these themes they found teachers ultimately made the final decision on what materials were used, and lack of time and funding for the curriculum were barriers to implementation (18). Process evaluation of the Pathways program, a prevention trial to promote physical activity and healthy eating in American Indian elementary school students, concurs with the findings of these other studies. Teacher comments identified snack preparation and taste testing as favorite student activities. The retrospective and self-administered aspect of some of the process data collection was again cited as a limitation, and in the opinion of the researchers, the finding that many teachers left completion of this evaluation task until the end of the semester potentially introduced recall bias (19).

Our study, to our knowledge, is the first process evaluation of a service-oriented, rather than research-oriented, school-based nutrition and physical activity promotion effort comparable to state-initiated tobacco initiation prevention programs. Baranowski and Stables have suggested that the minimal components of a useful process analysis include examination of recruitment and retention of participants, context (environment), resources required, reach and exposure of the program, barriers, completeness and fidelity of implementation to the design of the program, continued use, and contamination (20). Although more typically used in scripted interventions, many of these components are still qualifiable and quantifiable within the character of LAUSDNN.

## Methods

### Data sources

The data were consolidated from three sources: district online school demographics (5), LAUSDNN administrative records, and the Nutrition Network End-Year Report, a satisfaction survey of the entire program administered to lead teachers. The survey was sent by school mail to the lead teacher of participating schools at the end of the 2001–2002 school year. Responses were voluntary. Each returned survey was read by the same investigator, and answers were sorted into recurrent themes and then tallied for comparison. A few schools returned completed surveys from school administrators other than the lead teacher; however, scoring and analysis were done only for the lead teacher responses.

### Outcome measures

The study evaluated LAUSDNN by examining four key process evaluation indicators: recruitment, retention, program highlights, and program barriers.

### Independent variables

Obesity prevalence is higher among Latino and African American children and youth than among whites. Thus, the main independent variable of interest to us was school ethnic composition. The ethnic composition of participating schools was based on LAUSD survey data, in which student ethnicity was determined by parent identification or by the personal observation of teachers. District-wide ethnic composition of schools was derived from LAUSD (7). In the evaluation of retention factors, included as independent variables in addition to ethnic composition of schools were the percentage of students offered free and reduced school meals, whether or not the school had an action grant, and school enrollment and grade level (elementary vs all other grades).

### Analysis

We conducted a retrospective cross-sectional study, using the secondary data, of the elements of the LAUSDNN implementation process in the 2001–2002 school year with individual LAUSDNN schools as the unit of analysis. Retention rates were evaluated as the reapplication to the program in the 2002–2003 school year.

Recruitment of schools into the program for 2001–2002 was analyzed with a bivariate comparison, comparing ethnic composition of schools with LAUSD district-wide data. We analyzed the reapplication of schools for the next school year (2002–2003) in a multivariable framework. Frequencies are reported for program highlights and program challenges. In the multivariable analysis, we used hierarchical logistic regression to assess the associations of school characteristics with the outcome of a school reapplying to the program in the following year. Regressions were estimated as random effects models clustered by local school district. A hierarchical analysis was employed to account for unobserved factors that may have been related to the nested relationship of schools within their districts. For example, one school may have been more likely to participate because it was part of a district that had more investment in nutrition and health promotion activities.

We hypothesized that program retention and reapplication was influenced by the following characteristics: ethnic composition (i.e., white, Latino, African American, Asian American and Pacific Islander, American Indian, Alaska Native), action grant school status, percentage of students qualifying for free or reduced-price school lunches, and school grade levels (elementary vs all other schools). For any given ethnicity, a school with a percentage above the sample (participating schools) median for that ethnic group was assigned a 1; otherwise it was assigned a 0. The percentage of free and reduced-price lunch was also assessed as a dichotomous variable: above the median was assigned a 1, and below the median was assigned a 0. Elementary grade schools were assigned a 1; all other schools were grouped because of insufficient numbers and given a 0.

Odds ratios, *P* values, and 95% confidence intervals are reported for the multivariable analyses. Bivariate analyses were conducted using Microsoft Excel (Microsoft Corporation, Redmond, Washington), and multivariable models were estimated using STATA 8.0 (StataCorp LP, College Station, Texas).

## Results

### Recruitment and reapplication into the program

In the 2001–2002 school year, 574 schools, about 80% of the district K–12 schools, were eligible for the programs. Thirty-two percent (183/574) of these schools were recruited into full program participation, including an action grant (Table 1). Twenty-six additional schools participated in Harvest of the Month only, resulting in an overall 36% participation of the eligible schools at some level. The ethnic distribution of the combined student population of the LAUSDNN schools was similar to the district-wide profile, with 77% Latino and just over 10% African American students.

Nearly 80% (166/209) of the schools that participated in the program in the 2001–2002 school year reapplied to the program the next year. We compared the combined ethnic composition of the schools that continued from the 2001–2002 into the 2002–2003 school years with the schools that did not continue the program, and it was almost identical (data not shown).

Results of the logistic regression analysis assessing the association of school characteristics with the schools reapplying to LAUSDNN the following year (2002–2003) are presented in Table 2. We found that elementary schools were more than 3 times as likely as middle schools or high schools to reapply to LAUSDNN. Compared with schools that participated in Harvest of the Month only, schools that had action grants in addition to participating in Harvest of the Month had more than 3 times the odds of reapplying to LAUSDNN.

### Results from the *Nutrition Network End-Year Report* survey

Seventy-seven of the 183 schools returned lead teacher surveys (Table 3). Of surveys completed, 69% (53/77) of respondents indicated Harvest of the Month as the program highlight, followed by school salad bar at 17% (13/77), using grant funds at 12% (9/77), and school garden at 12% (9/77).

Challenges cited by survey respondents are presented by frequency in Table 3. The process of making purchases with grant money (e.g, ordering food and supplies) was cited in 36% (28/77) of the surveys. Survey comments indicated a lack of familiarity with this process, dissatisfaction with the fixed quantities of supplied items (e.g., some items were only available in bulk necessitating extensive and time-consuming preparation by school staff), and perception that items were overpriced. Administering Harvest of the Month (i.e., receiving, division, classroom delivery of the produce) was cited as a challenge by 21% (16/77) of respondents. Required documentation was cited as a challenge by 18% (14/77).

## Discussion

Our study suggests several lessons from a process evaluation of LAUSDNN, a service-oriented, school-based, nutrition and physical activity promotion effort. First, such an effort shows the potential in recruiting schools serving low socioeconomic status (SES) students of diverse ethnicity. Second, the relatively high retention rate in this voluntary program suggests that some program components were well-received despite challenges in administration and purchasing. Harvest of the Month was the most often cited highlight, possibly because it was more widely used than were other components. However, the favorable reviews on generating anticipation of produce delivery, offering a participatory experience for the students, and providing fresh produce attest to the strength of Harvest of the Month. Various school-initiated programs were also cited as highlights at the schools, reinforcing the notion that local choice was a vital part of LAUSDNN. Third, our evaluation of the factors associated with retention found that elementary schools were the most likely to reapply, suggesting that program components are attractive and perhaps more suitable in the elementary school setting. Fourth, SES gradations (as measured by the percentage of students eligible for free or reduced-price school meals) within these schools that already serve low-SES students did not matter when it came to retention. This suggests that retention may not be governed so much by constraints on school resources as by other factors, such as the degree of "local ownership" in a school. Fifth, and most importantly, the schools with a larger relative percentage of Latino and African American students were positively associated (Latino students, *P* = .08, African American Students, *P* = .13) with reapplying to LAUSDNN, indicating that schools that serve high proportions of these two ethnic groups, which are at high risk for overweight and obesity, can potentially be retained in voluntary demonstration programs.

A potential criticism of offering a voluntary program such as LAUSDNN in a diverse district is that only schools with a relatively high SES will have the time and support to take advantage of the enriching resources. Although ethnicity is an imperfect proxy for SES, African American and Latino children in Los Angeles County are several times more likely to live in poverty than are non-Latino whites, with 58% of African American children and 54% of Latino children living below 200% of the Federal Poverty Guidelines compared with 16% of non-Latino white children (21). Imposing the eligibility restriction means testing of having 50% or more students qualifying for a free or reduced-cost school lunch targeted the participation of families with lower SES and ensured that Latino and African American students were not disproportionately excluded.

We found that reapplying to the program was significantly positively associated with having an action grant versus the more passive component, participation in Harvest of the Month only. However, because the grant application mechanism was voluntary, the action grant's effect may be attributable to the greater local ownership of the program in schools that wrote grant proposals and operationalized their LAUSDNN-funded plan.

Insight into local-ownership issues is provided in studies of worksite nutrition interventions, which by circumstance have offered a degree of autonomy to participants similar to those in LAUSDNN. For example, the Working Well Trial, a large multicenter cancer prevention study, used an employee at each of the worksites as coordinator, and an employee advisory board was formed to plan and implement the core interventions. This was done to enhance participation, tailor core activities, and make institutionalization more likely (22). The trial found a significant increase in nutritional activities during the intervention that decreased significantly when the trial ended. The researchers concluded that maintenance might have been better if the advisory boards were not newly formed groups but rather were drawn from units with a shared mission, such as benefits or safety (23,24). The Treatwell 5-A-Day study also used a worksite coordinator and advisory board to tailor the program to meet the needs of the diverse ethnic groups at its worksites. The greatest improvement in diet in this study occurred in the arm of the trial that included families in the intervention (25). Also, later process analysis of this study provided evidence of a positive relationship between the number of events per employee, including advisory board-initiated activities, and fruit and vegetable consumption (26). The Seattle 5-a-Day worksite program phased in activities following the stages-of-change model but allowed the advisory board to tailor events to its worksite (27) and found a significant effect 2 years after baseline (28). The Arizona 5-a-Day worksite program used peer educators who received a stipend for their informal efforts of about 2 hours a week to alter health behavior norms. The peer educators continued their role even after the trial, indicating a degree of institutionalization. The number of fruits and vegetable servings consumed increased significantly, and consumption levels were maintained at 6 months following completion of the program (29).

These data must be interpreted in light of a number of limitations. First, our findings provide lessons for school districts with an ethnically diverse, low-income student population, so they may not be generalizable to school districts with a different composition of students. The program evaluation surveys suffered from flaws that led to a low response rate and may have introduced stakeholder bias. Reducing respondent burden could increase the response rate. For example, the two surveys could be combined and simplified by replacing open-ended questions with Likert-scale ratings for highlights and challenges, now that major categories have been established. Targeting the lead teachers reduced the potential pool of respondents and emphasized the person at the school who had a tremendous investment in the program.

This process analysis of LAUSDNN indicates that schools serving low SES students of diverse ethnicity can be recruited into and retained in a large nutrition and physical activity program. The effectiveness of the program and the ability to institutionalize it in schools might be positively affected by fostering local ownership, that is, allowing the school personnel who apply for the grant to tailor the program to their schools in order to promote healthy dietary choices and physical activity among their students.

## Figures and Tables

**Table 1:** Recruitment of Eligible Schools (N = 574) by Major Program Components, Los Angeles Unified School District Nutrition Network, Los Angeles, California, 2001–2002

Program Component	Description	No. of Participating Schools	Recruitment Rate, %
Action grants	Grant money awarded to operate a school-originated nutrition and physical activity plan	183	32
Harvest of the Month	Produce delivered to schools for sampling	209	36
Harvest of the Month Newsletter	Newsletter suggests educational activities related to monthly produce	209	36
Chefs in the Classroom	Professional area chefs demonstrate healthful meals	81	14
Nutrition advisory councils^a^	Students plan positive changes in school environment	77	13
School gardening	Specialists provide workshops, organize donated supplies	114	20

a Nutrition advisory councils received a different action award, which the schools had to apply for separately. Because of resource constraints, many schools elected not to go through this additional application process and did not apply.

**Table 2:** Characteristics of Schools (N = 209) and Likelihood of Reapplying to the Los Angeles Unified School District Nutrition Network, Los Angeles, California, 2002–2003

Characteristic	OR (95% CI^a^)	*P* Value
Percentage of white students at school is above median value^b^	1.21 (0.48-3.02)	.68
Percentage of Latino students at school is above the median value^b^	2.72 (0.90-8.21)	.08
Percentage of African American students at school is above median value^b^	2.22 (0.78-6.30)	.13
Percentage of AAPI students at school is above median value^b^	1.04 (0.11-9.55)	.93
Percentage of American Indian/Alaska Native students at school is above median value^b^	1.00 (1.00-1.00)	.99
Percentage of students at school eligible for free and reduced-price meals is above median value^b^	1.00 (1.00-1.00)	.99
School has action grant (vs school with no action grant)	3.53 (1.50-8.33)	.004
Elementary school (vs middle or high school)	3.23 (1.43-7.32)	.005

OR indicates odds ratio; CI, confidence interval; AAPI, Asian American and Pacific Islander.

a CIs reflect standard error adjustment resulting from clustering by local school district.

b Median value refers to the percentage of students calculated for all schools participating in the study (n = 209). Referent group is schools with an at-median or below-median percentage.

**Table 3:** Los Angeles Unified School District Nutrition Network, Highlights and Challenges Cited in Surveys (N = 77), Los Angeles, California, 2002

**Program Highlights**	Frequency cited
Harvest of the Month	53
School salad bar	13
Using grant funds	9
School garden	9
Fair	8
Mural	8
Cook in classroom	8
Nutrition advisory council	6
5-a-Day materials	3

**Program Challenges**	**Frequency cited**

Ordering food and supplies	28
Administering Harvest of the Month	16
Required documentation	14
Cooking and preparing food	10
Lack of support at school	10
Administering salad bars	8
Lack of support of Food Services	6
Adding physical activity	4
Other	9
